# OralNet: Fused Optimal Deep Features Framework for Oral Squamous Cell Carcinoma Detection

**DOI:** 10.3390/biom13071090

**Published:** 2023-07-07

**Authors:** Ramya Mohan, Arunmozhi Rama, Ramalingam Karthik Raja, Mohammed Rafi Shaik, Mujeeb Khan, Baji Shaik, Venkatesan Rajinikanth

**Affiliations:** 1 Department of Computer Science and Engineering, Saveetha School of Engineering, Saveetha Institute of Medical and Technical Sciences, Chennai 602105, India; ramyanallu@saveetha.com (R.M.); sriramkarthik83@gmail.com (R.K.R.); v.rajinikanth@ieee.org (V.R.); 2 Department of Chemistry, College of Science, King Saud University, P.O. Box 2455, Riyadh 11451, Saudi Arabia; kmujeeb@ksu.edu.sa; 3 School of Chemical Engineering, Yeungnam University, Gyeongsan 38541, Republic of Korea; shaikbaji@yu.ac.kr

**Keywords:** oral cancer, OSCC, VGG16, DenseNet201, OralNet, classification

## Abstract

Humankind is witnessing a gradual increase in cancer incidence, emphasizing the importance of early diagnosis and treatment, and follow-up clinical protocols. Oral or mouth cancer, categorized under head and neck cancers, requires effective screening for timely detection. This study proposes a framework, OralNet, for oral cancer detection using histopathology images. The research encompasses four stages: (i) Image collection and preprocessing, gathering and preparing histopathology images for analysis; (ii) feature extraction using deep and handcrafted scheme, extracting relevant features from images using deep learning techniques and traditional methods; (iii) feature reduction artificial hummingbird algorithm (AHA) and concatenation: Reducing feature dimensionality using AHA and concatenating them serially and (iv) binary classification and performance validation with three-fold cross-validation: Classifying images as healthy or oral squamous cell carcinoma and evaluating the framework’s performance using three-fold cross-validation. The current study examined whole slide biopsy images at 100× and 400× magnifications. To establish OralNet’s validity, 3000 cropped and resized images were reviewed, comprising 1500 healthy and 1500 oral squamous cell carcinoma images. Experimental results using OralNet achieved an oral cancer detection accuracy exceeding 99.5%. These findings confirm the clinical significance of the proposed technique in detecting oral cancer presence in histology slides.

## 1. Introduction

The incidence of cancer in the human population is steadily increasing due to various factors, necessitating the need for appropriate screening and diagnosis to enable timely detection and treatment. Recent literature has confirmed that cancer rates are rising among individuals regardless of age, race and sex, leading to the development and implementation of numerous awareness programs and clinical protocols aimed at reducing the impact of the disease [1,2,3].

According to the 2020 report by the World Health Organization (WHO), cancer is responsible for 10 million deaths worldwide. The report also highlights that the low- and lower-middle-income countries account for approximately 30% of cancer cases caused by infections such as human papillomavirus (HPV) and hepatitis. Early detection and effective treatment have the potential to cure many types of cancer, leading to the development of various clinical protocols for cancer detection and assessment of its severity [4].

The Global Cancer Observatory’s (Globocan2020) report for 2020 provides comprehensive information on new cancer cases and cancer-related deaths globally. It presents country-wise and gender-wise statistics regarding cancer-related deaths. Recent research suggests that oral cancer (OC), a type of cancer affecting the lip and oral cavity, ranks 16th in terms of its occurrence and death rates globally. Early detection and treatment are pivotal in achieving complete remission, particularly in regions such as Asia, where the incidence of OC is significantly higher (65.8% of global cases) with a death rate of approximately 74%. Notably, the use of tobacco is identified as a major causal factor for OC [5].

The clinical diagnosis of the OC involves several steps, including symptom analysis, personal examination by a clinician, medical image-assisted detection and confirmation of cancer severity through a biopsy test. Microscopic analysis plays a crucial role in identifying the stage and severity of oral squamous cell carcinoma (OSCC), the most common type of oral cancer worldwide.

In the recent literature, researchers commonly employ microscopy images for the detection of OSCC, often utilizing machine-learning (ML) and deep-learning (DL) techniques [6,7,8]. This proposed research aims to develop a DL-assisted diagnosis system called OralNet using microscopic images provided by Rahman et al. [9]. The dataset used for this study consists of H&E-stained tissue slides collected, prepared and catalogued by medical experts. The slides were obtained from 230 patients using a Leica ICC50 HD microscope. The dataset contains two categories of images: 100× magnification (89 healthy and 439 OSCC) and 400× magnification (201 healthy and 495 OSCC) [10]. For this work, 1500 RGB-scaled images were extracted through image cropping, resulting in 1500 healthy and 1500 OSCC images for the proposed DL approach.

The developed OralNet consists of the following stages: (i) Image collection, cropping and resizing, (ii) deep-features extraction using pretrained models, (iii) handcrafted feature extraction, (iv) feature optimization using the artificial hummingbird algorithm (AHA) and (v) binary classification using a three-fold cross validation. Each pretrained DL model employed in this study generates one-dimensional (1D) features, features of size 1 × 1 × 1000, providing comprehensive information about the normal and OSCC images. Additionally, handcrafted features such as local binary pattern (LBP) with various weights and discrete wavelet transform (DWT) are combined with the deep features to improve detection accuracy in OC detection with OralNet.

In this study, OralNet is separately implemented on the histology images at 100× and 400× magnifications, and the results are presented and discussed. OralNet utilizes the following classification approaches: (i) individual deep features (DF), (ii) dual-deep features (DDF), (iii) ensemble deep features (EDF), (iv) DF + HF, (v) DDF + HF and (vi) EDF + HF. The achieved results are compared and verified. The experimental outcome demonstrates that using DDF + HF achieves a detection accuracy of over 99.5% when employing classifiers such as SoftMax, decision-tree (DT), random-forest (RF) and support-vector-machine (SVM) with a linear kernel for both 100× and 400× magnified histology slides. Additionally, the K-nearest neighbors (KNN) classifier achieves 100% detection accuracy with the chosen image database.

The proposed OralNet framework, utilizing DL and ML techniques, demonstrates high accuracy in detecting OSCC in microscopic images, making it clinically significant. It holds promise for future applications in examining H&E-stained tissue slides obtained from the cancer clinics.

This research work focuses on the development of the OralNet framework and makes several significant contributions, including:

a. Verification and confirmation of the performance of pretrained DL schemes in detecting OSCC on H&E-stained tissue slides: The study validates the effectiveness of various pretrained DL models in accurately identifying OSCC in histology slides.
b. Enhancement of OSCC detection performance through the combination of deep features with local binary pattern (LBP) and discrete wavelet transform (DWT): By integrating handcrafted features such as LBP and DWT with deep features, the research improves the overall accuracy and effectiveness of OSCC detection.
c. Feature optimization using the artificial hummingbird algorithm (AHA): The study utilizes AHA to identify the optimal combination of deep and handcrafted features, leading to improved performance in detecting OSCC.
d. Classification using individual, serially fused and ensemble features: The research explores different approaches to feature combinations and evaluates their performance for OSCC detection. This includes utilizing individual deep features, fusing them sequentially with handcrafted features and constructing ensemble features to achieve optimal classification results.

The major contributions of this research work involve validating pretrained DL schemes for OSCC detection, enhancing detection performance through feature combination, optimizing features using AHA and evaluating the performance of various feature fusion and ensemble techniques in OSCC classification.

This research work is divided into several sections. Section 2 details the methodology and implementation of the proposed OralNet framework. Section 3 and Section 4 present the experimental results and conclude the research, respectively.

Automatic disease diagnosis has become a standard practice in modern healthcare and the effectiveness of automated diagnostic systems largely relies on the quality and diversity of the disease dataset used for training. When utilizing a clinical database, it becomes possible to develop and implement a diagnostic scheme that performs well in real clinical settings.

With the increasing incidence rates of cancer, there is a growing need for improved diagnostic accuracy. Machine learning (ML) and deep learning (DL) techniques have been proposed and applied to enhance cancer diagnosis. In this research, the focus is on oral cancer (OC), which is a prevalent oral health issue globally, particularly in Asia. While various computerized methods have been developed for cancer diagnosis using medical imaging, DL-supported approaches have shown greater efficiency in achieving higher accuracy.

Table 1 provides a summary of selected OC detection methods reported in the literature, highlighting the different techniques and their respective performances in detecting OC.

In a recent study by Alab et al. [22], a comprehensive review of oral cancer (OC) detection using various computer algorithms was conducted. The findings of this research confirmed that previous works have achieved detection accuracies of up to 100%. Additionally, a recent deep learning (DL) study by Das et al. [21] demonstrated the clinical significance of DL-based OSCC detection, highlighting the need for a new DL scheme to assist doctors in OC diagnosis. Further, a few recent works also demonstrate the image supported detection of the OSCC [23,24]. Motivated by these findings, the proposed research aims to develop a novel scheme called OralNet for the detection of cancer in histology slides. To improve the accuracy of the detection, this work incorporates a combination of deep and handcrafted features optimized with the artificial hummingbird algorithm (AHA). By integrating these techniques, the study aims to achieve enhanced accuracy and contribute to the field of OC diagnosis.

## 2. Materials and Methods

This section of the research focuses on implementation of the proposed OralNet scheme, which involves stages ranging from image resizing to classification. The main objective of OralNet is to classify histology slides into healthy or oral squamous cell carcinoma (OSCC) classes, considering both 100× and 400× magnifications. The subsections within this part of the study describe the construction of OralNet and its evaluation using the selected performance metrics.

### 2.1. OralNet Framework

**Figure 1:**
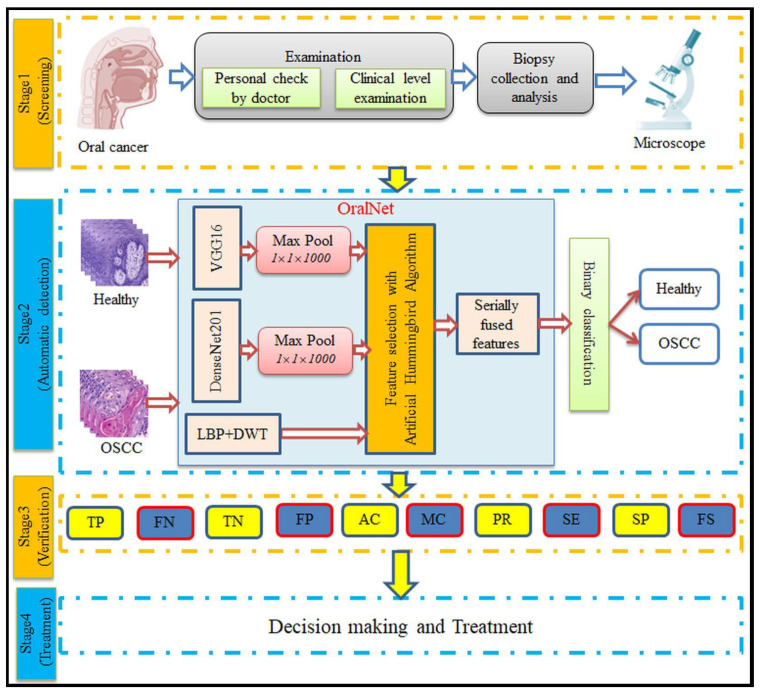
Developed scheme to detect the OC using the histology slides.

Figure 1 illustrates the proposed framework for oral cancer (OC) detection, depicting the various stages involved in the disease detection process.

Stage 1 represents the initial screening phase, where an experienced clinician performs a personal examination to identify any oral abnormalities. This is followed by confirmation using a specific clinical protocol. If abnormalities are detected, biopsy samples are collected from the affected area, and microscopic images are obtained using a digital microscope at a chosen magnification level. These images are then used for further analysis to determine the presence and severity of the cancer.

Stage 2 focuses on the implementation of the proposed OralNet scheme for automatic cancer detection. Firstly, the acquired images are resized to a predetermined level. Then, relevant features are extracted using a combination of deep learning techniques and handcrafted approaches. To reduce the dimensionality of the extracted features, an optimized feature reduction technique called LBA (artificial hummingbird algorithm) is applied. The reduced features are then concatenated sequentially to form a new one-dimensional (1D) feature vector. This feature vector plays a crucial role in effectively classifying the images into healthy and OSCC classes, resulting in improved performance metrics.

Stage 3 evaluates the performance of the proposed approach based on the obtained performance metrics. The confirmed OSCC diagnosis and its severity are documented in a report, which is shared with the healthcare professional responsible for planning and implementing the appropriate treatment using recommended clinical procedures.

The presented framework encompasses screening, automatic detection, verification and treatment stages, providing a comprehensive approach for OC detection and management.

The proposed OralNet in this research combines deep and handcrafted features to achieve accurate classification of oral histology images into healthy and OSCC categories. One of the key strengths of this scheme is its ability to handle images captured at both 100× and 400× magnifications, ensuring improved detection accuracy regardless of the magnification level. By utilizing the artificial hummingbird algorithm (LBA) to optimize and serially concatenate features from VGG16, DenseNet201 and the handcrafted feature extraction process, the proposed scheme achieves a remarkable detection accuracy of 100% when employing the K-nearest neighbors (KNN) classifier.

### 2.2. Image Database

**Figure 2:**
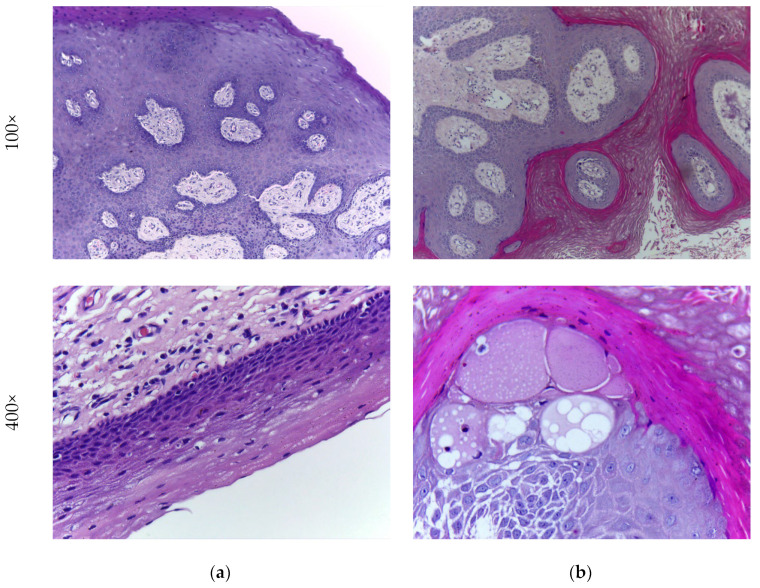
Sample H&E-stained histology slides of healthy and OSCC category. (**a**) Healthy; (**b**) OSCC.

In order to validate the clinical significance of the computerized disease detection procedure, it is crucial to utilize a dataset consisting of histology slides collected from real patients. In this study, the OC dataset obtained from [10], which comprises 1224 H&E-stained histology slides captured using a Leica ICC50 HD microscope (Leica, Wetzlar, Germany), is employed for assessment. The dataset includes 518 images recorded at 100× magnification and 696 images captured at 400× magnification. Each image has a pixel dimension of 2048 × 1536 × 3 pixels. It is worth noting that this dataset contains a larger number of OSCC slides compared to healthy histology slides. For further details about this database, reference can be made to the work conducted by Rahman et al. [9]. Figure 2 illustrates a sample image from each class, with Figure 2a representing a healthy histology slide and Figure 2b displaying an OSCC slide.

### 2.3. Test Image Generation

The DL-assisted disease detection using the medical images is crucial for accurate and timely diagnosis, reducing the burden on healthcare professionals. However, computerized image examination procedures have limitations and require preprocessed images as input. Image resizing is a critical step in the computerized disease diagnosis process to ensure compatibility with the algorithms used.

**Figure 3:**
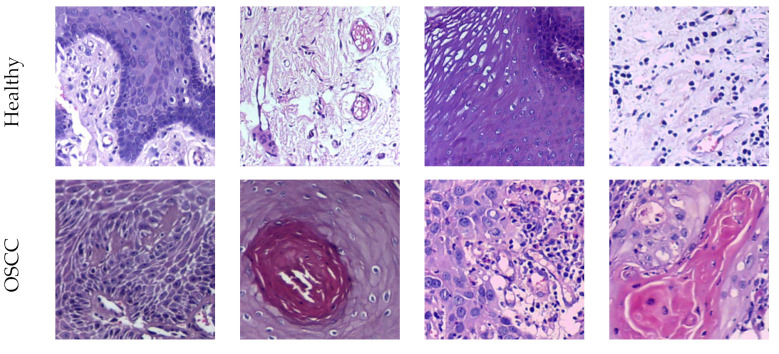
Generated test images from 100× magnified microscopy slide.

**Figure 4:**
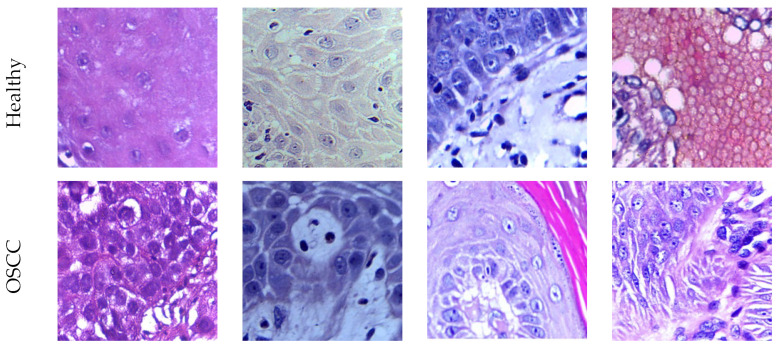
Generated test images from 400× magnified microscopy slide.

The proposed scheme in this research utilizes pretrained DL methods which require the image to be resized to a specified pixel value (224×224×3). The raw histology slides are first subjected to cropping and resizing to obtain the necessary test images for extracting deep and handcrafted features. In this process, image sections without vital information are discarded. Following this procedure, a total of 1500 histology slides in the healthy/OSCC class are obtained for both the 100× and 400× magnified images. These images are then utilized to evaluate the performance of the developed OralNet scheme. Figure 3 showcases the histology slides collected using a 100× microscopy image, while Figure 4 displays the images derived from the raw images magnified at 400×. These images serve as the basis for evaluating the performance of the OralNet scheme.

### 2.4. Feature Extraction and Reduction

The accuracy of automatic data analysis using computerized algorithms relies heavily on the information contained within the selected database and the mining procedures applied to extract relevant features. These mined features from the medical dataset are then used to train and evaluate the performance of the implemented computer algorithm for automatic disease detection. To prevent overfitting, feature reduction techniques are employed, and the performance of the developed scheme is assessed using a 3-fold cross validation.

Recent research in the field has demonstrated that integrating deep features and handcrafted features leads to improved detection accuracy in automatic disease detection. In the proposed OralNet scheme, the integration of deep and handcrafted features is utilized to enhance classification accuracy. Additionally, to mitigate the risk of overfitting, feature optimization based on the AHA (adaptive harmony search) algorithm is implemented, reducing the number of image features considered in the detection process.

#### 2.4.1. Deep-Features Mining

The key features from the selected histology images are extracted using pretrained deep learning (PDL) methods. These PDL schemes are computer programs specifically designed for tasks in the medical imaging domain, such as recognizing specific types of medical images, detecting abnormalities and making predictions about a patient’s health. PDL schemes are valuable tools for healthcare professionals as they enable quick and accurate identification of abnormalities in medical images, aiding in informed decision-making and treatment planning.

In this study, several PDL schemes were considered, including VGG16, VGG19, ResNet18, ResNet50, ResNet101 and DenseNet201. Detailed information about these schemes can be found in the literature [25,26,27,28,29]. Each PDL approach produces a one-dimensional (1D) feature vector of size 1 × 1 × 1000, which is utilized to evaluate the classifier’s performance in categorizing the images into healthy and OSCC classes.

#### 2.4.2. Handcrafted Features Mining

In the field medical image processing, the use of handcrafted features in machine learning-based image classification tasks is well-established [30,31,32]. Recent studies in medical image classification have shown that integrating deep features with handcrafted features leads to improved diagnostic accuracy compared to using deep features alone [33,34,35]. Handcrafted features such as local binary patterns (LBP) [36,37] and discrete wavelet transform (DWT) are commonly employed by researchers in medical image classification tasks [38,39,40]. These features are combined with the deep features to enhance disease detection performance.

**Figure 5:**
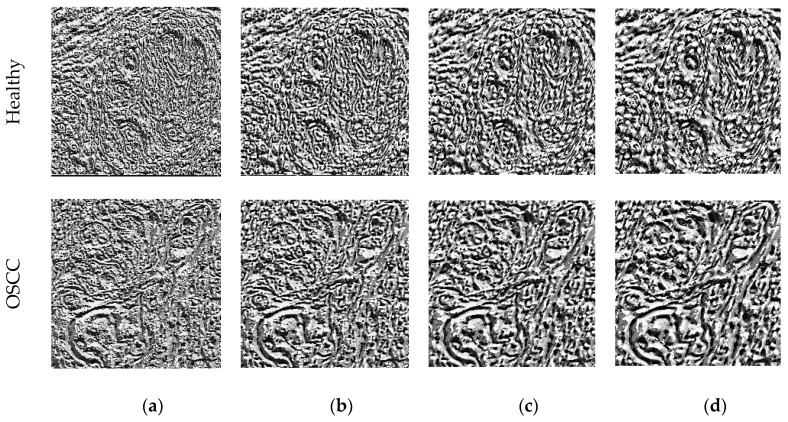
LBP images achieved for W = 1 to 4. (**a**) W = 1; (**b**) W = 2; (**c**) W = 3; (**d**) W = 4.

In this research, the weighted LBP method proposed by Gudigar et al. [41] was employed to extract LBP features. The weights used in the LBP calculation ranged from 1 to 4 (W = 1 to 4). The resulting LBP patterns for healthy and OSCC images are shown in Figure 5a–d representing different weight values. Each LBP pattern generates a 1D feature vector of size 1 × 1 × 59, which is expressed in Equations (1)–(4). The overall LBP feature vector is represented by Equation (5).

(1) $${{LBP}_{w1}}_{\left(1\times 1\times 59\right)}={{LBP}_{1}}_{\left(1,1\right)},{{LBP}_{1}}_{\left(1,2\right)},\ldots ,{{LBP}_{1}}_{\left(1,59\right)}$$

(2) $${{LBP}_{w2}}_{\left(1\times 1\times 59\right)}={{LBP}_{2}}_{\left(1,1\right)},{{LBP}_{2}}_{\left(1,2\right)},\ldots ,{{LBP}_{2}}_{\left(1,59\right)}$$

(3) $${{LBP}_{w3}}_{\left(1\times 1\times 59\right)}={{LBP}_{3}}_{\left(1,1\right)},{{LBP}_{3}}_{\left(1,2\right)},\ldots ,{{LBP}_{3}}_{\left(1,59\right)}$$

(4) $${{LBP}_{w4}}_{\left(1\times 1\times 59\right)}={{LBP}_{4}}_{\left(1,1\right)},{{LBP}_{4}}_{\left(1,2\right)},\ldots ,{{LBP}_{4}}_{\left(1,59\right)}$$

(5) $${LBP}_{\left(1\times 1\times 236\right)}={{LBP}_{w1}}_{\left(1\times 1\times 59\right)}+{{LBP}_{w2}}_{\left(1\times 1\times 59\right)}+{{LBP}_{w3}}_{\left(1\times 1\times 59\right)}+{{LBP}_{w4}}_{\left(1\times 1\times 59\right)}$$

**Figure 6:**
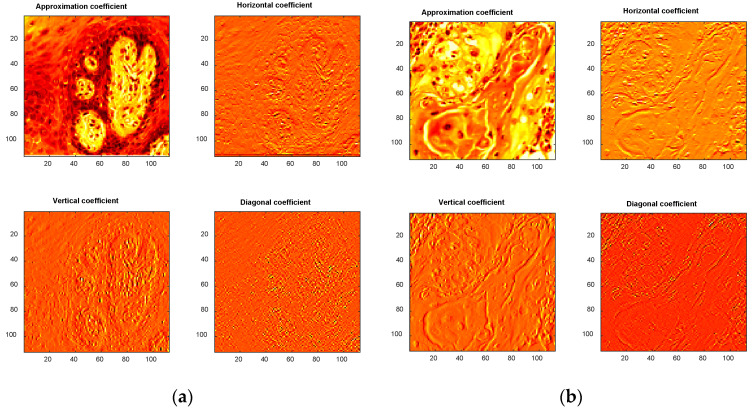
The DWT patterns achieved for a chosen image. (**a**) Healthy; (**b**) OSCC.

In addition to *LBP*, this study also incorporated *DWT* features. The *DWT* scheme was applied to each test image, resulting in the image being decomposed into four components: approximate, vertical, horizontal and diagonal coefficients, as illustrated in Figure 6. Figure 6a,b depicts the corresponding outcomes for the healthy and OSCC categories, respectively, represented using a hot color map. From each image, a 1D feature vector of size 1 × 1 × 45 was extracted, as shown in Equations (6)–(9). The complete *DWT* feature vector is represented by Equation (10). The handcrafted features utilized in this research are a combination of the *LBP* and *DWT* features, as expressed in Equation (11).

(6) $${{DWT}_{approximate}}_{\left(1\times 1\times 45\right)}={{DWT}_{1}}_{\left(1,1\right)},{{DWT}_{1}}_{\left(1,2\right)},\ldots ,{{DWT}_{1}}_{\left(1,45\right)}$$

(7) $${{DWT}_{vertical}}_{\left(1\times 1\times 45\right)}={{DWT}_{2}}_{\left(1,1\right)},{{DWT}_{2}}_{\left(1,2\right)},\ldots ,{{DWT}_{2}}_{\left(1,45\right)}$$

(8) $${{DWT}_{horizontal}}_{\left(1\times 1\times 45\right)}={{DWT}_{3}}_{\left(1,1\right)},{{DWT}_{3}}_{\left(1,2\right)},\ldots ,{{DWT}_{3}}_{\left(1,45\right)}$$

(9) $${{DWT}_{diagonal}}_{\left(1\times 1\times 45\right)}={{DWT}_{4}}_{\left(1,1\right)},{{DWT}_{4}}_{\left(1,2\right)},\ldots ,{{DWT}_{4}}_{\left(1,45\right)}$$

(10) $${DWT}_{\left(1\times 1\times 180\right)}={{DWT}_{1}}_{\left(1\times 1\times 45\right)}+{{DWT}_{2}}_{\left(1\times 1\times 45\right)}+{{DWT}_{3}}_{\left(1\times 1\times 45\right)}+{{DWT}_{4}}_{\left(1\times 1\times 45\right)}$$

(11) $${Handcrafted\;features}_{\left(1\times 1\times 416\right)}={LBP}_{\left(1\times 1\times 236\right)}+{DWT}_{\left(1\times 1\times 180\right)}$$

#### 2.4.3. Hummingbird Algorithm for Feature Optimization

The artificial hummingbird algorithm (AHA) procedure was developed based on artificially mimicked foraging behaviors in hummingbirds (HB) [42]. When searching for food sources (flowers), HBs take into account various factors such as flower type, nectar quality, refill rate and previous visits. In the AHA optimization exploration, each flower represents a solution vector, and the nectar replenishing rate serves as the fitness value for the algorithm. The AHA is initiated with assigned values for the HBs and the flowers (food sources). The performance of the AHA is monitored using a visit table that keeps track of the number of visits by HBs to each food source. Food sources that receive more visits are considered more valuable and are given higher priority for nectar collection [43,44,45].

**Figure 7:**
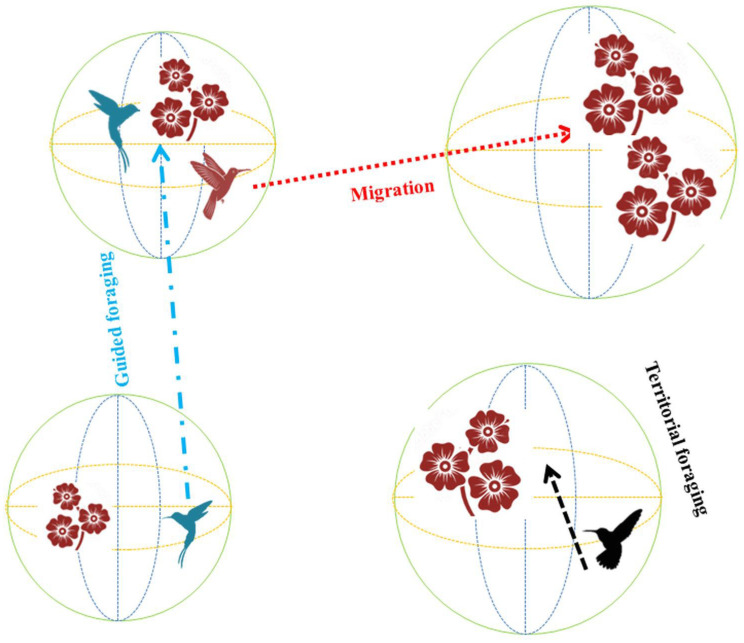
Hummingbird algorithm activity in the search space.

The artificial hummingbird algorithm (AHA) classifies hummingbirds (HB) into three distinct foraging patterns: territorial, guided and migration, as depicted in Figure 7. These foraging patterns involve three-dimensional searches conducted by HBs in specific regions using different flight paths such as axial flight, diagonal flight and omnidirectional flight. The primary goal of HBs during their foraging activities is to efficiently locate the optimal solution for a given problem by employing these diverse three-dimensional search strategies.

##### Initialization

(12) $$X_{i}=L+ℜ\left(U-L\right)\;for\;i=j=1,2,\ldots ,n$$

where ℜ = random vector [0,1], L = lower limit, U = upper limit, i = quantity of flowers and $X_{i}$ = position of the *i*th flower.

The visit-table created in AHA is depicted below;

(13) $${VT}_{i,j}={\{}_{null\;i=j}^{0\;if\;i\neq j}$$

where ${VT}_{i,j}$ represents the HB’s visit to a specific flower to collect the nectar.

##### Guided Foraging

During this process, the HB is allowed to visit the flower that contains the highest volume of nectar and ${VT}_{i,j}$ is considered to locate the flower. When identifying the appropriate food, the HB will perform different flight patterns as shown in the following diagram:

(14) $$Axial\;flight=D^{\left(i\right)}={\{}_{0\;else}^{1\;if\;i=randi(\left[1,d\right])}\;\mathrm{for}\;i=1,2,\ldots ,d$$

where *d* = search space and $randi(\left[1,d\right])$ = formation of random number of value 1 to *d*.

(15) $$Diagonal\;flight=D^{\left(i\right)}={\{}_{0\;else}^{1\;if\;i=P\left(j\right),j\in \left[1,k\right],P=randperm\left(k\right),k\in [2,\left[ℜ*\left(d-2\right)\right]+1]}$$

where $randperm\left(k\right)$ = random permutation of integers from 1 to *k*

(16) $$Omnidirectional\;flight=D^{\left(i\right)}=1$$

The guided foraging is mathematically as follows:

(17) $$V_{i}\left(t+1\right)=X_{i,tar}\left(t\right)+(a\times D\times \left(X_{i}\left(t\right)-X_{i,tar}\left(t\right)\right))$$

(18) a~N(0,1)

where $X_{i}\left(t\right)$ = position of the *i*th flower in a chosen time (*t*), $X_{i,tar}\left(t\right)$ = target flower and a = guiding parameter computed using normal distribution (*N*) having mean = 0 and standard deviation = 1.

The position update for HD towards *i*th flower is;

(19) $$X_{i}\left(t+1\right)={\{}_{V_{i}\left(t+1\right)\;f\left(X_{i}\left(t\right)\right)\;>\;f(V_{i}\left(t+1\right)}^{X_{i}(t)\;f(X_{i}\left(t\right))\;\leq \;f(V_{i}\left(t+1\right))}$$

where *f* = fitness, which specifies the flower with better nectar-refilling rate.

##### Territorial Foraging

After consuming nectar from a target flower, the hummingbird (HB) tends to prioritize searching for new food sources rather than revisiting familiar flowers. In the territorial foraging process, the HB will explore and move to other available flowers within its current location to gather additional food. This behavior reflects the HB’s tendency to maximize its foraging efficiency by seeking out new opportunities for nourishment;

(20) $$V_{i}\left(t+1\right)=X_{i}\left(t\right)+(b\times D\times X_{i}\left(t\right))$$

(21) b~N(0,1)

Here *b* = territorial factor computed using normal distribution (*N*) having mean = 0 and standard deviation = 1.

##### Migration Foraging

When the food supply within a territory is depleted, the hummingbird (HB) will initiate migration behavior and move to a more distant location in search of a suitable new food source. During this process, the HB will travel over longer distances, expanding its search range to locate the desired food source. This migration behavior allows the HB to explore new areas and increase its chances of finding abundant and replenished food sources.

(22) $$X_{worst}(t+1)=L+R\left(U-L\right)$$

where $X_{worst}(t+1)$ = new position of the HB when the food source becomes the worst (lack of nectar).

#### 2.4.4. Serial Features Concatenation

In this subsection, the feature optimization technique using the artificial hummingbird algorithm (AHA) and serial feature concatenation is presented. The AHA parameters are set as follows: the number of HBs (hummingbirds) is N = 25, the search dimension is D = 2, the maximum number of iterations is Iter_max = 2500 and the stopping criteria are based on the maximization of the Cartesian distance (CD) between features or reaching the maximum number of iterations.

**Figure 8:**
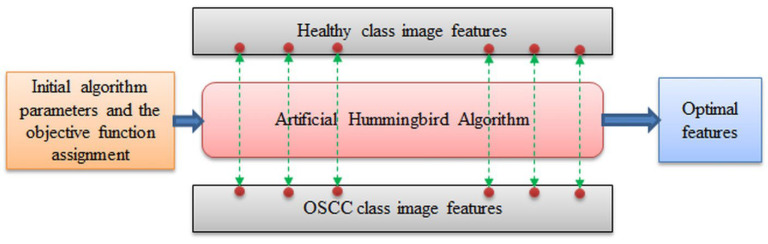
Optimal feature selection using AHA.

The AHA optimization process aims to find the individual features that are most relevant for distinguishing between healthy and OSCC samples based on the CD. The AHA algorithm helps in identifying the optimal features by iteratively exploring the feature space. The optimization and serial concatenation process is illustrated in Figure 8.

Once the optimal features are determined, a new 1D feature vector is generated by concatenating these features in a sequential manner. This concatenated feature vector is then utilized to evaluate the performance of the proposed OC detection scheme. The effectiveness of the feature optimization and serial concatenation approach is verified by comparing the detection results with previous studies [46,47].

The proposed work utilizes the artificial hummingbird algorithm (AHA) to identify the optimal values of deep and handcrafted features. The AHA helps in reducing the feature space and selecting the most discriminative features for the detection of oral cancer. These reduced features are then combined to form a new one-dimensional (1D) feature vector.

By integrating the reduced features, the proposed scheme aims to improve the performance of oral cancer detection. The new 1D feature vector captures the essential information from both the deep and handcrafted features, providing a comprehensive representation of the histology images. This combined feature vector is then used to evaluate the effectiveness of the proposed scheme in accurately detecting oral cancer. The utilization of AHA for feature optimization and the subsequent combination of reduced features into a 1D feature vector contribute to enhancing the performance of the proposed scheme for oral cancer detection.

### 2.5. Performance Evaluation and Validation

To validate the performance of the OralNet system, it is crucial to evaluate it using clinical-grade datasets, as this helps establish the significance of the oral squamous cell carcinoma (OSCC) detection system at the developmental stage. In this study, a dataset consisting of 3000 test images (1500 healthy and 1500 OSCC) was utilized to assess the effectiveness of the developed OralNet, considering both 100× and 400× magnification images. The true-positive (TP) and true-negative (TN) images, representing the actual healthy and OSCC categories, were used for validation.

In cases where the implemented scheme detects false-positive (FP) or false-negative (FN) values in addition to TP and TN, these values are used to construct a confusion matrix and calculate various performance metrics. These metrics include accuracy (AC), misclassification (MC), precision (PR), sensitivity (SE), specificity (SP) and F1-score (FS), which are essential for assessing the validity of the implemented scheme. The mathematical notations for these measures can be found in Equations (23)–(28) in the literature [48,49].

Furthermore, these measures are computed independently for each classifier, including SoftMax, decision-tree (DT), random-forest (RF), K-nearest neighbors (KNN) and support-vector machine (SVM) with a linear kernel [50,51]. Additionally, receiver operating characteristic (ROC) curves are constructed based on sensitivity and specificity, which serve as a means to further verify the validity of the method. The achieved accuracy demonstrates the superiority of the proposed scheme, thereby confirming its clinical importance. The performance of the OralNet system is validated using clinical-grade datasets, and various performance metrics, including accuracy and ROC curves, supporting the effectiveness and clinical significance of the proposed scheme in detecting OSCC.

(23) $$AC=\frac{TP+TN}{TP+TN+FP+FN}\times 100$$

(24) MC=100−AC

(25) $$PR=\frac{TP}{TP+FP}\times 100$$

(26) $$SE=\frac{TP}{TP+FN}\times 100$$

(27) $$SP=\frac{TN}{TN+FP}\times 100$$

(28) $$FS=\frac{2TP}{2TP+FN+FP}\times 100$$

### 2.6. Implementation

The developed OralNet system was implemented on a workstation with the following specifications: Intel i5, 16 GB RAM and 4 GB VRAM. Python 3.11.2 was used as the programming language for executing the work. The results obtained from each technique were individually presented and discussed. The prime focus of this study was on the deep features obtained through the pretrained deep learning (PDL) schemes, which served as the key information for the disease detection task.

For the classification task, 80% of the data (2400 images) was used for training, 10% (300 images) for validation and the remaining 10% (300 images) for testing, following a 3-fold cross-validation approach. The parameters assigned for these schemes were as follows: learning rate of 1 × 10^−5^, Adam optimization, max pooling, ReLU activation, a total of 1500 iterations, total epochs of 150 and SoftMax as the default classifier.

The experimental investigation considered different combinations of deep features (DF), deep and handcrafted features (DDF, EDF) and their ensemble with handcrafted features (DF + HF, DDF + HF, EDF + HF). The performance was evaluated based on computed metrics for both 100× and 400× histology slides. Initially, DF-based classification was implemented using a 1D feature vector of size 1 × 1 × 1000. Based on the achieved classification accuracy, DenseNet201 was ranked as the top-performing PDL approach, followed by VGG16 and ResNet101, for both 100× and 400× image categories. The ensemble of these three PDL features was considered as EDF, and its optimized value was used for EDF + HF. Furthermore, the AHA optimized features of VGG16 and DenseNet201 were serially concatenated to obtain DDF.

The computation of EDF in this work was based on the approach proposed by Kundu et al. [52]. The selection of EDF was done by considering performance measures such as accuracy (AC), precision (PR), sensitivity (SE), specificity (SP) and F1-score (FS) of VGG16, ResNet101 and DenseNet201, as depicted in Equations (29)–(31) in the literature. The developed OralNet system was implemented on a workstation with specific specifications, and the performance of various PDL approaches and their combinations with handcrafted features was evaluated. The selection of the best-performing features was based on the computed performance measures, ensuring the optimal performance of the system for both 100× and 400× histology slides.

(29) $$A^{i}=({AC}_{i},{PR}_{i},{SE}_{i},{SP}_{i},{FS}_{i})$$

Computation of the ensemble probability score is presented below;

(30) $${ens}_{j}=\frac{\sum_{i}w^{\left(i\right)}\times {p_{j}}^{\left(i\right)}}{\sum_{i}w^{\left(i\right)}}$$

where $w^{\left(i\right)}=\sum_{x\epsilon A^{i}}tanh(x)$

(31) $${prediction}_{j}=argmax({ens}_{j})$$

The AHA-based optimization helps to obtain optimal values of VGG16 (1×1×371), DenseNet201 (1×1×416), HF (1×1×103) and EDF (1×1×366). These features are then serially integrated to obtain other feature vectors as shown in Equations (32)–(34).

(32) $${DDF}_{\left(1\times 1\times 787\right)}={VGG16}_{\left(1\times 1\times 371\right)}+{DenseNet201}_{\left(1\times 1\times 416\right)}$$

(33) $${\left(DDF+HF\right)}_{\left(1\times 1\times 890\right)}={DDF}_{\left(1\times 1\times 787\right)}+{HF}_{\left(1\times 1\times 103\right)}$$

(34) $${\left(EDF+HF\right)}_{\left(1\times 1\times 469\right)}={EDF}_{\left(1\times 1\times 366\right)}+{HF}_{\left(1\times 1\times 103\right)}$$

## 3. Result and Discussions

**Figure 9:**
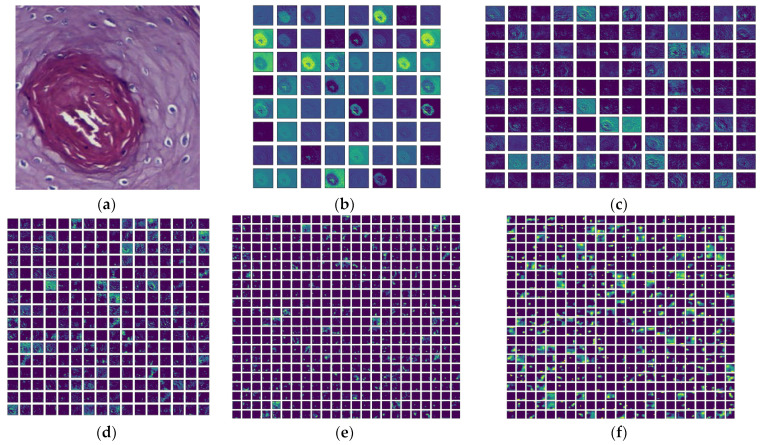
Convolutional layer outcomes of VGG16 for a chosen OSCC test image. (**a**) Test image; (**b**) Convolution1; (**c**) Convolution2; (**d**) Convolution3; (**e**) Convolution4; (**f**) Convolution5.

**Figure 10:**
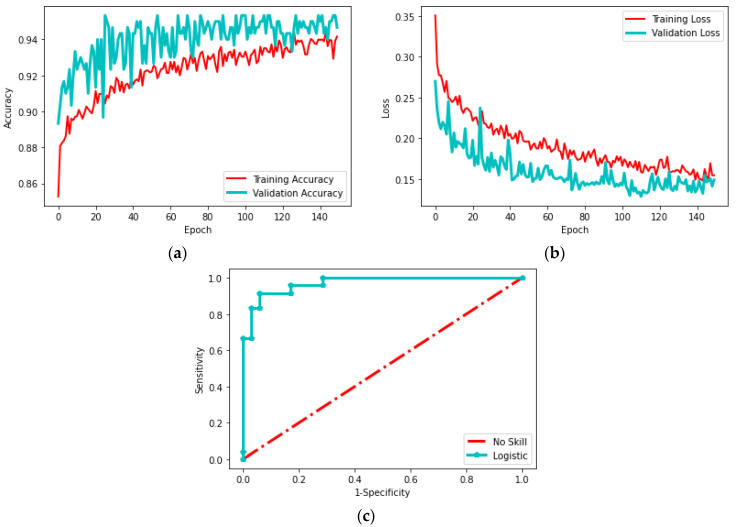
The result achieved during the training and testing operation on VGG16. (**a**) Accuracy; (**b**) Loss; (**c**) RoC curve.

This section presents the experimental results obtained from the proposed work on the oral cancer (OC) histology image database for binary classification using three-fold cross-validation. The chosen pretrained deep learning (PDL) schemes were analyzed on the histology image database at 100× magnification. Each PDL was trained for 150 epochs, and the best result from the three-fold cross-validation was selected for evaluation. The VGG16 scheme was used for classification, and the outcome is illustrated in Figure 9. Figure 9a shows a test image, while Figure 9b–f depicts the results of various convolutional layers using the Viridis color map. These images demonstrate the transformation of the test image into features as it passes through the layers of the VGG16 scheme, resulting in a 1D feature vector of size 1 × 1 × 1000. The accuracy, loss and ROC curve achieved with this process are presented in Figure 10. Figure 10a,b shows the training and validation accuracy and loss, respectively, while Figure 10c displays the ROC curve with an area under the curve of 0.957, confirming the improved classification accuracy achieved by VGG16.

**Figure 11:**
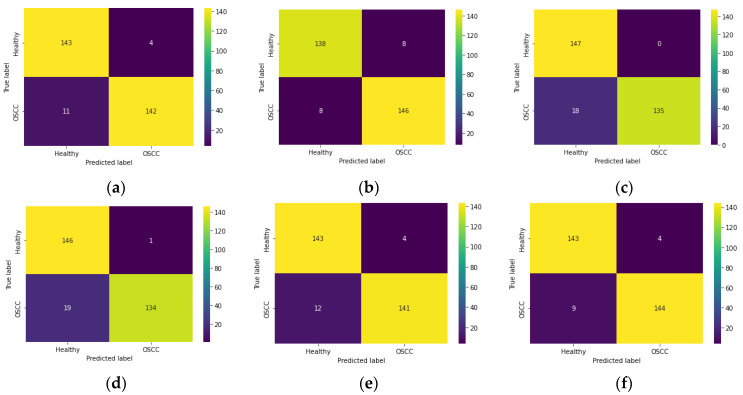
Confusion matrix obtained during the classification of 100× histology images. (**a**) VGG16; (**b**) VGG19; (**c**) ResNet18; (**d**) ResNet50; (**e**) ResNet101; (**f**) DenseNet201.

The effectiveness of this scheme is further confirmed using a confusion matrix (CM), which provides important measures such as true positives (TP), true negatives (TN), false positives (FP) and false negatives (FN). Using these values, additional metrics including accuracy (AC), misclassification (MC), precision (PR), sensitivity (SE), specificity (SP), and F1-score (FS) are computed. Figure 11 presents the CM obtained with various PDL schemes using the SoftMax classifier. Figure 11a shows the CM for VGG16, while Figure 11b–f depicts the CM for other PDL schemes with the SoftMax classifier.

The performance metrics obtained from the CM are computed and presented in Table 2 for both 100× and 400× magnified histology slides. The table demonstrates that PDL schemes such as VGG16, ResNet101 and DenseNet201 achieve higher classification accuracy compared to VGG19, ResNet18 and ResNet50. These top-performing schemes are then used to obtain deep and handcrafted features (DDF and EDF) after possible feature reduction with the artificial hummingbird algorithm (AHA), as discussed in Section 2.6.

**Figure 12:**
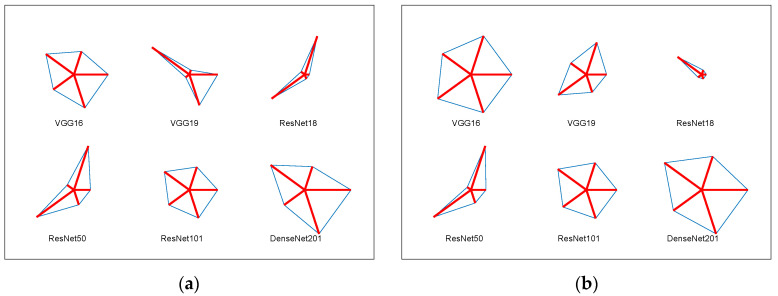
Glyph plot to confirm the overall merit of the considered PDL scheme. (**a**) 100×; (**b**) 400×.

The overall performance of the selected PDL schemes is further verified using the glyph plot, as shown in Figure 12. This plot confirms that DenseNet201 and VGG16 are ranked 1st and 2nd, respectively, based on their achieved classification accuracy. Figure 12a,b displays the glyph plots for 100× and 400× images, respectively.

Once the performance of VGG16 with the SoftMax classifier was verified, its effectiveness was further evaluated using other classifiers such as DT, RF, KNN and SVM, as shown in Table 3. For the 100× image database, the SoftMax classifier exhibited superior results compared to the other methods. However, for the 400× images, the KNN classifier achieved higher accuracy compared to the other methods, including the SoftMax classifier. A similar evaluation process was conducted for DenseNet201, and the results are presented in Table 4. This table confirms that the KNN classifier outperformed other classifiers for both the 100× and 400× images in terms of classification accuracy.

Table 5 displays the results obtained for the DDF-based classification of the selected OC histology images. It demonstrates that the KNN classifier achieves better accuracy for the 100× histology slides. In the case of 400× histology images, both DT and KNN classifiers exhibit higher accuracy compared to the other classifiers employed in this study.

Table 6 presents the classification results obtained using EDF. It confirms that the KNN classifier yields better accuracy for the 100× images. For the 400× images, the accuracy achieved with the RF and KNN classifiers is comparable and superior to that of the SoftMax, DT and SVM classifiers. The results presented in Table 5 and Table 6 indicate that the classification accuracy is generally higher for the 100× images compared to the 400× images.

The results of the classification task using the integrated deep and handcrafted features (DDF + HF) are presented in Table 7. The table confirms that the KNN classifier achieves a detection accuracy of 100% for both 100× and 400× images. Additionally, other classifiers also achieve a detection accuracy of over 98.5%, demonstrating the effectiveness of the proposed OralNet in detecting oral cancer from the histology slides.

The performance of the integrated ensemble deep and handcrafted features (EDF + HF) is evaluated using the selected database, and the results are presented in Table 8. The table shows that the considered feature vector enables achieving a classification accuracy of over 99% for each classifier in the chosen image datasets. This further confirms that the EDF + HF approach provides a higher detection accuracy for the given database.

**Figure 13:**
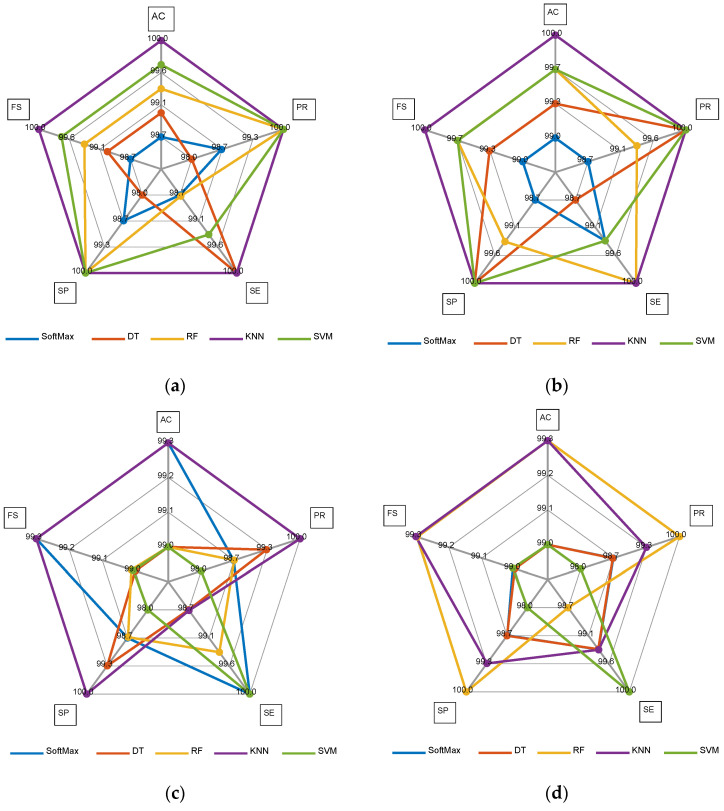
Overall performance evaluation of the OralNet using the spider plot for the chosen histology database. (**a**) DDF + HF (100×); (**b**) DDF + HF (400×); (**c**) EF + HF (100×); (**d**) EF + HF (400×).

To visualize the overall performance of the chosen classifiers, Table 7 and Table 8 are represented graphically using a spider plot in Figure 13. Figure 13a,b presents the results for DDF + HF with 100× and 400× images, respectively, highlighting the effectiveness of the KNN classifier in detecting OSCC. Figure 13c,d depicts the outcomes achieved with EDF + HF, indicating that the classification accuracy of this approach is also high and comparable to DDF + HF for both image cases.

This proposed research work introduces the novel OralNet scheme for improved examination of OC histology images with higher accuracy. The evaluation of this scheme is conducted using 100× and 400× magnified microscopy images, and the results obtained validate the effectiveness of the proposed approach in achieving better detection accuracy when employing serially concatenated deep and handcrafted features. The limitation of this study is that the performance of the outcome may change based on the dimension of the data and the training hyperparameter.

In the future, this scheme holds potential for evaluation of clinically collected OC histology images. By applying the OralNet approach to real-world data, its performance and reliability can be further assessed, contributing to the development of an advanced and clinically relevant OC detection system.

## 4. Conclusions

Oral cancer is a critical medical condition, and early detection and treatment are crucial for successful outcomes. Biopsy-supported diagnosis, which involves microscopic examination of histology slides, is a common clinical procedure for confirming the presence and severity of cancer. This research focused on the analysis of microscopic images taken at 100× and 400× magnification to develop a novel OralNet scheme for examining and classifying healthy and OSCC (oral squamous cell carcinoma) images. The main objective of this study was to implement a binary classifier with a three-fold cross-validation technique to accurately classify the chosen image dataset. Various feature vectors were considered, and the integrated deep and handcrafted features (DDF + HF) demonstrated superior detection accuracy compared to other feature combinations explored in this research. The dataset used for assessment consisted of 3000 images, with an equal distribution of 1500 healthy and 1500 OSCC samples. The experimental results of the proposed EDF + HF approach yielded a classification accuracy of over 99%, showcasing its effectiveness in accurately identifying healthy and OSCC images. The DDF + HF-based classification also exhibited excellent performance, with the KNN classifier achieving a remarkable 100% accuracy. Furthermore, the proposed OralNet scheme outperformed similar existing works in the literature in terms of classification accuracy. These findings strongly support the effectiveness of the DDF + HF-based approach for oral cancer detection using histology images. In future research, it would be valuable to validate and assess the performance of the proposed scheme with clinically collected histology slides, providing an opportunity to evaluate its effectiveness in real-world scenarios.

## Figures and Tables

**Table 1:** Summary of automatic oral cancer detection methods.

Procedure and Outcome	Reference
In this study, a 12-layer deep convolutional neural network (CNN) was implemented to perform the segmentation of oral squamous cell carcinoma (OSCC) from the selected histology slides. The proposed CNN architecture was specifically designed to accurately identify and delineate the boundaries of OSCC regions within the slides. The experimental results demonstrated a segmentation accuracy exceeding 97%, indicating the effectiveness of the CNN-based approach in accurately segmenting OSCC from histology slides.	[11]
In this scheme, an ensemble deep features (EDF) approach was utilized in combination with the empirical wavelet transform feature for the detection of oral squamous cell carcinoma (OSCC) and oral cancer (OC). The EDF method incorporates multiple deep learning features to enhance detection accuracy. Through the integration of the empirical wavelet transform feature, which captures relevant information from the input data, the scheme achieved a detection accuracy of 92%. This demonstrates the efficacy of the proposed approach in accurately identifying OSCC and OC cases.	[12]
The implementation of AlexNet, a popular deep learning architecture, was employed to detect oral squamous cell carcinoma (OSCC) images from the selected database in this study. By utilizing the AlexNet model, the research achieved an impressive accuracy of 97.66% in accurately identifying OSCC cases. The results indicate the effectiveness of the implemented AlexNet model in accurately detecting and distinguishing OSCC images within the database.	[13]
In this study, a deep transfer learning approach was utilized to detect oral squamous cell carcinoma (OSCC) images from histology images magnified at 100× and 400×. By leveraging transfer learning techniques, the model was able to leverage knowledge from pretrained networks to enhance its performance in OSCC detection. Using ensemble features, the implemented approach achieved high detection accuracies of 98% for 100× magnified images and 96% for 400× magnified images. These results demonstrate the effectiveness of the deep transfer learning approach in accurately identifying OSCC cases in different magnifications of histology images.	[14]
The detection of oral squamous cell carcinoma (OSCC) from histopathological images using deep learning (DL) techniques was examined in this study. By combining the features extracted from VGG16, InceptionV3 and ResNet50 models, a classification accuracy of 97% was achieved. This highlights the effectiveness of utilizing a fusion of DL features from different models for accurate OSCC detection in histopathological images. The results demonstrate the potential of DL-supported methods in improving the accuracy of OSCC classification and enhancing the diagnostic capabilities of oral cancer detection systems.	[15]
In this study, an automatic detection scheme for oral squamous cell carcinoma (OSCC) from histology images using machine learning (ML) techniques was introduced. By incorporating morphological and texture features and employing the DT classifier, the scheme achieved an impressive detection accuracy of 99.78%. This demonstrates the efficacy of utilizing ML-based approaches in accurately identifying OSCC cases from histology images. The inclusion of morphological and texture features enhances the discriminatory power of the classifier, leading to highly accurate detection results.	[16]
In this research, a machine learning (ML)-based approach was employed for the detection of oral squamous cell carcinoma (OSCC). The detection scheme utilized histogram and grey-level co-occurrence matrix features. By incorporating principal component analysis (PCA)-based feature generation, the proposed method achieved a remarkable detection accuracy of 100%. This highlights the effectiveness of the ML approach in accurately identifying OSCC cases using extracted features derived from the histogram and grey-level co-occurrence matrix. The utilization of PCA for feature generation further enhanced the accuracy of the detection process.	[17]
In this study, transfer learning with a convolutional neural network (CNN) was employed to classify histology images. By leveraging the knowledge and pretrained weights from an existing CNN model, the implemented transfer learning approach achieved a high classification accuracy of 97.50%. This demonstrates the effectiveness of transfer learning in leveraging pre-existing CNN architectures to improve the accuracy of histology image classification. The results highlight the potential of utilizing transfer learning techniques for the accurate and efficient classification of histology images in various medical applications.	[18]
In this research, a convolutional neural network (CNN) was utilized for the automatic classification of oral cancer (OC) images. By implementing the CNN architecture, the study achieved an impressive classification accuracy of 96.77% in distinguishing between healthy and oral squamous cell carcinoma (OSCC) images. This highlights the effectiveness of CNN-based methods in accurately classifying OC images and differentiating between healthy and cancerous samples. The results demonstrate the potential of CNNs as a valuable tool in the automatic detection and classification of OC, aiding in early diagnosis and improved patient outcomes.	[19]
Using a transfer learning scheme, this research implemented a detection method for oral cancer (OC) based on capsule networks. The capsule network architecture demonstrated its efficacy in accurately detecting OC, achieving a binary accuracy of 97.35%. By leveraging pretrained weights and knowledge from existing models, the transfer learning approach enhanced the performance of the capsule network in classifying OC images. These findings highlight the potential of capsule networks and transfer learning in improving the accuracy of OC detection, offering promising prospects for enhancing diagnostic capabilities in oral cancer screening.	[20]
In this study, a 10-layer deep learning (DL) scheme was implemented for the detection of oral squamous cell carcinoma (OSCC) from histology images. The proposed DL scheme achieved a high detection accuracy of 97.82%. By leveraging the multi-layer architecture, the DL model effectively learned and extracted discriminative features from the histology images, enabling the accurate identification of OSCC cases. The results highlight the potential of DL techniques in improving the detection and diagnosis of OSCC, contributing to more efficient and reliable screening processes in clinical settings.	[21]
This research provides a comprehensive review of oral cancer (OC) detection using a variety of machine learning (ML) and deep learning (DL) techniques. The study focuses on analyzing a clinical database and thoroughly discusses the findings. The results of the research demonstrate the effectiveness of computerized schemes in accurately analyzing and interpreting clinical data associated with OC. By leveraging ML and DL procedures, the study highlights the potential of these approaches in improving the detection and diagnosis of OC. The comprehensive analysis of the clinical database reinforces the significance of computerized methods in enhancing our understanding and management of OC, contributing to more effective and efficient healthcare practices.	[22]

**Table 2:** Classification results achieved with PDL schemes with SoftMax classifier.

Image	Scheme	TP	FN	TN	FP	AC	MC	PR	SE	SP	FS
100×	VGG16	142	11	143	4	95.0000	5.0000	97.2603	92.8105	97.2789	94.9833
	VGG19	146	8	138	8	94.6667	5.3333	94.8052	94.8052	94.5205	94.8052
	ResNet18	134	19	146	1	93.3333	6.6667	99.2593	87.5817	99.3197	93.0556
	ResNet50	135	18	147	0	94.0000	6.0000	100	88.2353	100	93.7500
	ResNet101	141	12	143	4	94.6667	5.3333	97.2414	92.1569	97.2789	94.6309
	DenseNet201	144	9	143	4	95.6667	4.3333	97.2973	94.1176	97.2789	95.6811
400×	VGG16	141	7	144	8	95.0000	5.0000	94.6309	95.2703	94.7368	94.9495
	VGG19	139	10	142	9	93.6667	6.3333	93.9189	93.2886	94.0397	93.6027
	ResNet18	140	8	138	14	92.6667	7.3333	90.9091	94.5946	90.7895	92.7152
	ResNet50	139	13	141	7	93.3333	6.6667	95.2055	91.4474	95.2703	93.2886
	ResNet101	141	7	142	10	94.3333	5.6667	93.3775	95.2703	93.4211	94.3144
	DenseNet201	143	5	143	9	95.3333	4.6667	94.0789	96.6216	94.0789	95.3333

**Table 3:** Evaluating the performance of the VGG16 with different binary classifiers.

Dimension	Classifier	TP	FN	TN	FP	AC	MC	PR	SE	SP	FS
100×	SoftMax	142	11	143	4	95.0000	5.0000	97.2603	92.8105	97.2789	94.9833
	DT	142	6	142	10	94.6667	5.3333	93.4211	95.9459	93.4211	94.6667
	RF	144	7	138	11	94.0000	6.0000	92.9032	95.3642	92.6174	94.1176
	KNN	140	9	144	7	94.6667	5.3333	95.2381	93.9597	95.3642	94.5946
	SVM	141	10	143	6	94.6667	5.3333	95.9184	93.3775	95.9732	94.6309
400×	SoftMax	141	7	144	8	95.0000	5.0000	94.6309	95.2703	94.7368	94.9495
	DT	142	10	143	5	95.0000	5.0000	96.5986	93.4211	96.6216	94.9833
	RF	141	7	142	10	94.3333	5.6667	93.3775	95.2703	93.4211	94.3144
	KNN	143	5	143	9	95.3333	4.6667	94.0789	96.6216	94.0789	95.3333
	SVM	142	9	143	6	95.0000	5.0000	95.9459	94.0397	95.9732	94.9833

**Table 4:** Evaluating the performance of the DenseNet201 with different binary classifiers.

Dimension	Classifier	TP	FN	TN	FP	AC	MC	PR	SE	SP	FS
100×	SoftMax	144	9	143	4	95.6667	4.3333	97.2973	94.1176	97.2789	95.6811
	DT	143	8	140	9	94.3333	5.6667	94.0789	94.7020	93.9597	94.3894
	RF	142	9	144	5	95.3333	4.6667	96.5986	94.0397	96.6443	95.3020
	KNN	144	8	144	4	96.0000	4.0000	97.2973	94.7368	97.2973	96.0000
	SVM	143	4	143	10	95.3333	4.6667	93.4641	97.2789	93.4641	95.3333
400×	SoftMax	143	5	143	9	95.3333	4.6667	94.0789	96.6216	94.0789	95.3333
	DT	141	8	143	8	94.6667	5.3333	94.6309	94.6309	94.7020	94.6309
	RF	142	10	143	5	95.0000	5.0000	96.5986	93.4211	96.6216	94.9833
	KNN	144	3	143	10	95.6667	4.3333	93.5065	97.9592	93.4641	95.6811
	SVM	144	5	142	9	95.3333	4.6667	94.1176	96.6443	94.0397	95.3642

**Table 5:** Evaluating the performance of DDF with different binary classifiers.

Dimension	Classifier	TP	FN	TN	FP	AC	MC	PR	SE	SP	FS
100×	SoftMax	146	3	146	5	97.3333	2.6667	96.6887	97.9866	96.6887	97.3333
	DT	146	6	145	3	97.0000	3.0000	97.9866	96.0526	97.9730	97.0100
	RF	147	4	144	5	97.0000	3.0000	96.7105	97.3510	96.6443	97.0297
	KNN	147	2	146	5	97.6667	2.3333	96.7105	98.6577	96.6887	97.6744
	SVM	143	6	147	4	96.6667	3.3333	97.2789	95.9732	97.3510	96.6216
400×	SoftMax	145	3	146	6	97.0000	3.0000	96.0265	97.9730	96.0526	96.9900
	DT	145	6	147	2	97.3333	2.6667	98.6395	96.0265	98.6577	97.3154
	RF	146	3	143	8	96.3333	3.6667	94.8052	97.9866	94.7020	96.3696
	KNN	146	7	146	1	97.3333	2.6667	99.3197	95.4248	99.3197	97.3333
	SVM	146	5	144	5	96.6667	3.3333	96.6887	96.6887	96.6443	96.6887

**Table 6:** Evaluating the performance of EDF with different binary classifiers.

Dimension	Classifier	TP	FN	TN	FP	AC	MC	PR	SE	SP	FS
100×	SoftMax	145	4	144	7	96.3333	3.6667	95.3947	97.3154	95.3642	96.3455
	DT	145	6	145	4	96.6667	3.3333	97.3154	96.0265	97.3154	96.6667
	RF	146	3	143	8	96.3333	3.6667	94.8052	97.9866	94.7020	96.3696
	KNN	145	6	146	3	97.0000	3.0000	97.9730	96.0265	97.9866	96.9900
	SVM	145	4	145	6	96.6667	3.3333	96.0265	97.3154	96.0265	96.6667
400×	SoftMax	146	4	143	7	96.3333	3.6667	95.4248	97.3333	95.3333	96.3696
	DT	145	4	144	7	96.3333	3.6667	95.3947	97.3154	95.3642	96.3455
	RF	144	7	146	3	96.6667	3.3333	97.9592	95.3642	97.9866	96.6443
	KNN	145	6	145	4	96.6667	3.3333	97.3154	96.0265	97.3154	96.6667
	SVM	143	6	146	5	96.3333	3.6667	96.6216	95.9732	96.6887	96.2963

**Table 7:** Evaluating the performance of DDF + HF with different binary classifiers.

Dimension	Classifier	TP	FN	TN	FP	AC	MC	PR	SE	SP	FS
100×	SoftMax	147	2	149	2	98.6667	1.3333	98.6577	98.6577	98.6755	98.6577
	DT	148	0	149	3	99.0000	1.0000	98.0132	100	98.0263	98.9967
	RF	149	2	149	0	99.3333	0.6667	100	98.6755	100	99.3333
	KNN	151	0	149	0	100	0.0000	100	100	100	100
	SVM	150	1	149	0	99.6667	0.3333	100	99.3377	100	99.6678
400×	SoftMax	148	1	149	2	99.0000	1.0000	98.6667	99.3289	98.6755	98.9967
	DT	150	2	148	0	99.3333	0.6667	100	98.6842	100	99.3377
	RF	149	0	150	1	99.6667	0.3333	99.3333	100	99.3377	99.6656
	KNN	150	0	150	0	100	1.0000	100	100	100	100
	SVM	148	1	151	0	99.6667	0.3333	100	99.3289	100	99.6633

**Table 8:** Evaluating the performance of EDF + HF with different binary classifiers.

Dimension	Classifier	TP	FN	TN	FP	AC	MC	PR	SE	SP	FS
100×	SoftMax	149	0	149	2	99.3333	0.6667	98.6755	100	98.6755	99.3333
	DT	148	2	149	1	99.0000	1.0000	99.3289	98.6667	99.3333	98.9967
	RF	150	1	147	2	99.0000	1.0000	98.6842	99.3377	98.6577	99.0099
	KNN	149	2	149	0	99.3333	0.6667	100	98.6755	100	99.3333
	SVM	149	0	148	3	99.0000	1.0000	98.0263	100	98.0132	99.0033
400×	SoftMax	149	1	148	2	99.0000	1.0000	98.6755	99.3333	98.6667	99.0033
	DT	148	1	149	2	99.0000	1.0000	98.6667	99.3289	98.6755	98.9967
	RF	149	2	149	0	99.3333	0.6667	100	98.6755	100	99.3333
	KNN	150	1	148	1	99.3333	0.6667	99.3377	99.3377	99.3289	99.3377
	SVM	149	0	148	3	99.0000	1.0000	98.0263	100	98.0132	99.0033

## Data Availability

Data contained within the article.
